# Engineered Human Tissue as A New Platform for Mosquito Bite-Site Biology Investigations

**DOI:** 10.3390/insects14060514

**Published:** 2023-06-02

**Authors:** Corey E. Seavey, Mona Doshi, Andrew P. Panarello, Michael A. Felice, Andrew K. Dickerson, Mollie W. Jewett, Bradley J. Willenberg

**Affiliations:** 1 Department of Internal Medicine, University of Central Florida College of Medicine, Orlando, FL 32827, USA; 2 Department of Mechanical, Aerospace, and Biomedical Engineering, Tickle College of Engineering, University of Tennessee, Knoxville, TN 37996, USA; 3 Division of Immunity and Pathogenesis, Burnett School of Biomedical Sciences, University of Central Florida College of Medicine, Orlando, FL 32827, USA

**Keywords:** Capgel, mosquitoes, *Aedes aegypti*, arthropods, blood-feeding

## Abstract

**Simple Summary:**

There is a dearth of in vitro tissue culture tools to study the complex biology of the skin bite site created by blood-feeding arthropods such as mosquitoes. To address this shortage, we engineered model human dermal microvascular bed tissue that included blood using capillary alginate gel (Capgel) biomaterial scaffolds and human cells. In a set of proof-of-concept experiments presented here, female *Aedes aegypti* bit into, probed, and blood-fed from these engineered dermal microvessel beds, similarly to how these mosquitoes would acquire blood meals from human hosts. Further, these tissue constructs remained intact and could be cleanly cultured for days after such blood meal acquisitions. Overall, the present study demonstrates this innovative new platform—termed a Biologic Interfacial Tissue-Engineered System (BITES)—with mosquitoes and signals its potential to break new ground in arthropod bite-site biology investigations.

**Abstract:**

Vector-borne diseases transmitted through the bites of hematophagous arthropods, such as mosquitoes, continue to be a significant threat to human health globally. Transmission of disease by biting arthropod vectors includes interactions between (1) saliva expectorated by a vector during blood meal acquisition from a human host, (2) the transmitted vector-borne pathogens, and (3) host cells present at the skin bite site. Currently, the investigation of bite-site biology is challenged by the lack of model 3D human skin tissues for in vitro analyses. To help fill this gap, we have used a tissue engineering approach to develop new stylized human dermal microvascular bed tissue approximates—complete with warm blood—built with 3D capillary alginate gel (Capgel) biomaterial scaffolds. These engineered tissues, termed a Biologic Interfacial Tissue-Engineered System (BITES), were cellularized with either human dermal fibroblasts (HDFs) or human umbilical vein endothelial cells (HUVECs). Both cell types formed tubular microvessel-like tissue structures of oriented cells (82% and 54% for HDFs and HUVECs, respectively) lining the unique Capgel parallel capillary microstructures. Female *Aedes* (*Ae*.) *aegypti* mosquitoes, a prototypic hematophagous biting vector arthropod, swarmed, bit, and probed blood-loaded HDF BITES microvessel bed tissues that were warmed (34–37 °C), acquiring blood meals in 151 ± 46 s on average, with some ingesting ≳4 µL or more of blood. Further, these tissue-engineered constructs could be cultured for at least three (3) days following blood meal acquisitions. Altogether, these studies serve as a powerful proof-of-concept demonstration of the innovative BITES platform and indicate its potential for the future investigation of arthropod bite-site cellular and molecular biology.

## 1. Introduction

Vector-borne diseases caused by pathogens such as parasites, bacteria, and viruses are responsible for more than 700,000 deaths annually [1]. Worldwide, major outbreaks of malaria, dengue virus (DENV), and Zika virus (ZIKV) transmitted by mosquitoes have caused overwhelming healthcare burdens. There are also more than 60 million cases of lymphatic filariasis caused by filarial parasites transmitted via mosquitoes [2,3,4]. These hematophagous (blood-feeding) arthropod vectors acquire the pathogens that cause these diseases during the ingestion of blood meals from infected vertebrate hosts. While probing for a blood vessel, a mosquito penetrates the stylet mouthparts of its proboscis through the vertebrate epidermis into the dermis and expectorates saliva along with the pathogen into the host skin [5,6]. For the continuation of the pathogen transmission cycle from vertebrate to mosquito, the pathogen must be abundant enough throughout the vertebrate body to be taken up again in the next mosquito bite [7]. In the orchestration of initial pathogen transmission, replication at the bite site and the early host immune responses are critical events prior to dissemination throughout the host [8,9]. Therefore, these early events are important for disease outcomes and strongly correlate with the peripheral pathogen burden and mortality [9]. 

In vitro tools such as artificial blood-feeding systems [10,11,12,13,14,15], cell cultures [16,17,18,19], and skin tissue explants [20,21] offer cost-effective means of maintaining closed-loop laboratory mosquito colonies, enable reductionist and/or high-throughput experimental approaches and have fewer ethical considerations and limitations compared to using live animals and human volunteers. However, none of these approaches allow the comprehensive analysis of mosquito biting/feeding and the molecular and cellular events that occur in the skin.

Artificial blood-feeding systems for maintaining mosquito colonies can be as straightforward as a warmed, blood-filled collagenous (bovine) sausage casing [11,12]. More sophisticated glass feeders, first designed by Rutledge et al. [13], that use Parafilm or animal-derived membranes (e.g., collagenous bovine casing, thinned chicken skin) stretched over a reservoir of blood warmed by a circulating water jacket are also frequently used [12,14]. Sri-in et al. developed a method using Parafilm^TM^-M membrane packets filled with warm blood for mosquito feeding and oral infection [15]. Using 3D printing, an acellular hydrogel-based “skin” with warmed blood circulating through vessel-like structures was recently developed for automated mosquito feeding and the study of repellents [22]. As all the above systems are acellular/lack living cells, they do not offer the potential for investigating cellular biology at the bite site.

Two-dimensional (i.e., flat) cell culture models have been informative as to the impacts of mosquito salivary proteins on human skin cells and have provided insights about arboviral infection in these cells [23]. Mosquitoes, however, cannot bite and blood-feed on 2D cell cultures and thus the important interplay between the vector mouthparts, expectorated saliva, transmitted pathogens (if any), and host cells (resident and migratory) at the skin bite site cannot be appreciated. Human ex vivo skin explants preserve the cellular and structural anatomy of the skin and have been used to examine dengue and Zika virus infections [20,21]. However, the supply and maintenance of these explants in culture are limited and mosquito bite/blood-feeding on the tissue has not been examined. Some of these shortcomings have been addressed through engineered full-thickness skin equivalents (FTSE) composed of collagen type 1 scaffolds with human keratinocytes and fibroblasts, but this model is avascular and does not include blood [24]. 

Together, all of the aforementioned gaps illustrate the need for in vitro-engineered 3D tissue platforms that mimic the skin/dermis with human cells and micro blood vessel structures into and from which arthropods—such as mosquitoes—can bite, probe, and blood-feed. Such tools would be powerful enablers of mosquito bite-site cellular and molecular biology investigations. Uniquely, to fill this need, we previously developed a versatile family of 3D self-assembled capillary alginate gel (Capgel) biomaterial scaffolds and studied these in a range of tissue engineering settings [25,26,27,28,29,30,31]. Then, in this proof-of-concept study, we have developed a Biologic Interfacial Tissue-Engineered System (BITES) that uses human cell-lined Capgel with blood loaded into the cellularized capillary lumens. This new in vitro platform was designed specifically to facilitate biological investigations of mosquito skin bite sites, and we demonstrated that female *Aedes (Ae.) aegypti* mosquitoes can bite, probe, and blood-feed naturally from BITES.

## 2. Materials and Methods

### 2.1. Mosquito Rearing

*Ae. aegypti* mosquitoes were reared as reported previously [32]. Briefly, 8 mg of eggs were brushed off germination paper and were added into a glass vial with 7.5 mL of larval food composed of 3% (g/mL) liver powder (MP Biologics, Santa Ana, CA, USA) and 2% (g/mL) brewer’s yeast (#1700, Insectrearing.com, Newark, DE, USA) in deionized (DI) water. Mosquito eggs and larval food were vigorously shaken and decanted into a tray of 3 L of DI water and incubated at 29–30 °C; this was considered day 0. On day 3, 7.5 mL of larval food was added to the larval rearing tray, followed by 10 mL on day 4 and day 5. On day 6, pouring/rinsing was performed over a 500 μm strainer and the larvae and pupae were transferred to a 50 cm^2^ surface area cup with 200 mL of DI water. The cup was kept in a rearing cage (8 × 8” Bioquip, Rancho Dominguez, CA, USA). Within 24 h after the rinse, the majority of the mosquitoes had emerged inside the cage. Finally, a cotton ball saturated with 10% sucrose (ThermoFisher, Waltham, MA, USA) was provided ad libitum as a food supply for the mature mosquitos by placing it on top of the rearing cage. On the day before experimentation, the sucrose cotton ball was removed and a cotton ball laden with DI water was provided ad libitum overnight.

### 2.2. Capgel Scaffold Synthesis and Processing

The synthesis of Capgel blocks was conducted as previously described [26]. Briefly, 10% bloom gelatin (G1890, Sigma-Aldrich, Saint Louis, MO, USA) was dissolved in distilled, deionized (ddH_2_O) water and degraded with sodium hydroxide (NaOH) at 80 °C for 72 h. An oligomeric 10% gelatin solution was equilibrated to ~23 °C for 2 h and mixed with a sodium alginate solution (Protanal Pharm Grade LF10/60, FMC Biopolymer, Philadelphia, PA, USA, supplied by IMCD US, LLC, Rochelle Park, NJ, USA) to create a 3% alginate, 2.6% gelatin solution. A 10 cm glass petri dish was prepared by coating its surfaces with 4% alginate dehydrated at 80 °C. The parent solution was then added to the alginate-coated dish and placed into a larger glass container. A copper (II) sulfate pentahydrate (CuSO_4_ 5H_2_O, Acros Organics, Flanders, Belgium) soaked Kimwipe was held taut by a plastic ring over the top of the petri dish, and an additional 0.1 M CuSO_4_ was then added dropwise to the surface of the Kimwipe for 10 min. The Kimwipe was then removed and the entire petri dish was submerged in 0.1 M CuSO_4_ for approximately 72 h. After the parent gel had grown, it was cut into strips and rinsed extensively over the following three days. The strips were then sectioned into ~5 × 5 × 3 mm blocks. 

Capgel blocks were crosslinked via carbodiimide chemistry as previously described [26]. Briefly, four ~5 × 5 × 3 mm Capgel blocks were added in a 50 mL conical tube, which was then filled with 20 mL of PBS containing 1.89 mg/mL of N-hydroxysuccinimide (ThermoFisher, Waltham, MA, USA). The conical tube was shaken gently overnight at 4 °C. Then, 20 mL of PBS containing 1.57 mg/mL N-(3-Dimethyl-aminopropyl)-N′-ethylcarbodiimide hydrochloride (Sigma-Aldrich, Saint Louis, MO, USA) was added to the conical tube, yielding a final reaction volume of 40 mL, which was gently shaken overnight at 4 °C. After completion of the crosslinking reaction, Capgel blocks were washed extensively for three to four days, with 0.2 μm filtered (564-0020, Nalgene, Rochester, NY, USA), sterile saline solution (0.9% NaCl, S271-3, ThermoFisher, Waltham, MA, USA). The rinses were repeated with filter-sterilized 10× sodium citrate solution (BP1325-4, ThermoFisher, Waltham, MA, USA), followed by another three rinses in sterile saline. Capgel blocks were then autoclaved using a liquid cycle and sterilization hold of 15 min. Sterilized Capgel blocks were then stored at 4 °C in sealed glass bottles.

### 2.3. Capgel Scaffold Cellularization

Capgel blocks were transferred to a 6-well plate (CytoOne, Ocala, FL, USA) with a sterile spatula (Corning Inc., Corning, NY, USA), in groups of four per well. To culture human dermal fibroblasts (HDF, CRL-2522, ATCC, Manassas, VA, USA), 5 mL of Dulbecco’s Modified Eagle Medium (DMEM, Gibco, ThermoFisher, Waltham, MA, USA) supplemented with 10% fetal bovine serum (FBS, Gibco, ThermoFisher, Waltham, MA, USA) and 1% penicillin–streptomycin (Gibco, ThermoFisher, Waltham, MA, USA) was used to submerge and culture HDF–Capgel blocks. To culture human umbilical vein endothelial cells (HUVECs, C0035C, Gibco, ThermoFisher, Waltham, MA, USA), 5 mL of Human Large Vessel Endothelial Cell Basal Medium 200 (Gibco, ThermoFisher, Waltham, MA, USA) supplemented with Large Vessel Endothelial Supplement (LVES, Gibco, ThermoFisher, Waltham, MA, USA) and 1% penicillin–streptomycin was used to submerge and culture HUVEC–Capgel blocks. The plate was then placed into a 37 °C, 5% CO₂ incubator (ThermoFisher Scientific Forma Series II Model 3110, Waltham, MA, USA) overnight. HDF and HUVEC cell lines were cultured in T-175 Cell Culture Flasks (Corning Inc., Corning, NY, USA) containing 25 mL of respective cell culture media. At 80% confluency, cells were incubated with 10 mL of 0.05% trypsin (Gibco, ThermoFisher, Waltham, MA, USA) for 5 min and spun down at 1905 relative centrifugal force (RCF) for 3 min (Thermo IEC Centra GP8R, 216 4-place swinging bucket rotor, ThermoFisher, Waltham, MA, USA) in a centrifuge. After centrifugation, the supernatant was removed, and the concentration of the resulting cell pellet was calculated using a phase counting chamber (Hausser Scientific, Horsham, PA, USA) and diluted to 40,000 live cells/µL. The media was then removed from the 6-well plate containing the Capgel blocks and a sterile gauze pad (4 × 4, 12-ply, ThermoFisher, Waltham, MA, USA) was used to remove excess media from the surface of the Capgel. One microliter of the cell pellet was then applied using a pipette tip to dispense and spread the cell suspension across the capillary openings of the Capgel. Capgels were placed with capillaries in the vertical direction in microcentrifuge tubes and briefly centrifuged. The cell-seeded Capgels were then placed into the incubator for 30 min to allow cell attachment. This process of seeding and incubating was repeated two more times so that a total of 3 µL of the cell pellets was seeded per Capgel block (120,000 cells/Capgel). Then, the Capgels were incubated for an additional 2 h to allow cell attachment, before adding 5 mL of media to each well, submerging the blocks. The cells were then cultured within the Capgel blocks for 4 weeks, undergoing a media change every two to three days.

### 2.4. Videography

In a rearing cage with one optically clear side (8 × 8” Bioquip, Rancho Dominguez, CA, USA), 20–50 *Ae. aegypti* female mosquitoes were sorted and sucrose-starved for ~18 h. The cage was placed under filming lights for 45 min so that the mosquitoes became acclimated to the lights and the room temperature before feeding (Figure S1A). A camera (Olympus Tough camera with an Ultimax 40.5 mm macro lens) was set in front of the clear side of the cage to record the blood-feeding events. A water bath (Isotemp 4100 H5P, ThermoFisher, Waltham, MA, USA) circulated warm water through an aluminum heat sink to regulate the temperature of the blood-loaded Capgel/BITES at 37 °C. The Capgel/BITES was placed on the heat sink immediately after blood loading in raftview orientation. Bare surfaces of the warm heat sink were covered with a thin piece of polystyrene foam insulation to localize heated areas to the Capgel/BITES and avoid diverting the mosquitos (Figure S1B).

### 2.5. Blood Meal Acquisition Experiments

After the mosquitoes were acclimatized to the videography setup, a Capgel block stored in saline solution at 4 °C or an HDF-cellularized Capgel block submerged in media at 37 °C was retrieved using a sterile spatula. The Capgel block was wiped on all sides with a sterile gauze pad for 30 s to remove excess saline/media and was placed in a petri dish. Using an inverted microscope (20×), the Capgel was placed on the petri dish with capillaries oriented in the vertical direction, and 10 µL of warm (37 °C) defibrinated bovine blood (HemoStat Laboratories, Inc., Dixon, CA, USA; the product was stored for less than two (2) weeks at 4 °C and used as received without modification) was slowly added on top of the capillaries, without dripping blood onto the external surfaces of the block. Blood was allowed to flow through the capillaries until erythrocytes were seen emerging from the other side of the Capgel using an inverted microscope. Next, with the sterile spatula, the Capgel was flipped 180° and 10 µL of blood was added on the opposite capillary ends. This process was repeated once more to achieve a final loaded blood volume of 40 µL in the Capgel/BITES. With blood loaded in the capillaries, the Capgel/BITES was transferred with sterile plastic forceps onto a sterile gauze pad with its capillaries horizontally disposed and carefully wiped with gauze on sides without capillary openings.

The HDF-cellularized BITES was presented as described above at different time intervals (4, 15, and 20 min) while recording the percentage of mosquitoes (*n* = 50) probing, biting, and/or blood-feeding every minute. To assess the blood meal duration and quantity of blood acquired, video recordings of mosquitoes (*n* = 50) acquiring blood from BITES were analyzed to assess changes in the abdomen width across time. Frames from the video were analyzed in ImageJ v1.53 (National Institute of Health, Bethesda, MD, USA) to calculate initial, intermediate, and final abdomen lengths by measuring the pleural membrane from tergum to sternum at the fourth abdominal segment. Measurements were scaled relative to the diameter of the head (0.67 mm). An abdomen width ratio was determined by the following equation:

*Abdomen Width Ratio = W_t_/W_i_* (1)

where *W_t_* is the abdomen width (mm) at a given time (s) during blood-feeding and *W_i_* is the abdomen width initially (i.e., prior to any ingestion/blood-feeding). To calculate the total volume of blood ingested, the volume of the abdomen was approximated as an ellipsoid, and mosquito abdominal dimensions were measured in ImageJ. The following equation was used to find the volume:

*Ellipsoid Volume = (4/3)πabc* (2)

where *a* is half the abdomen length taken from the abdomen–thorax border to the distal tip of the anus, *b* is half the abdomen width taken from tergum to sternum, and *c* is half the back of the mosquito across the midline and perpendicular to the long axis of the abdomen. The individual mosquito abdomen volume difference before and after blood feeding was used to calculate the blood volume ingested:

*∆V = Vf − Vi* (3)

where *V_f_* and *V_i_* are the final and initial abdomen volumes, respectively, and Δ*V* is the calculated blood volume ingested (µL). The volume of prediuretic excretion (i.e., urine) was approximated to the volume of a sphere:

*Sphere Volume = (4/3)πr^3^* (4)

where *r* is the radius of the prediuretic droplet measured using ImageJ. The calculated volume was then multiplied by the number of prediuretic droplets observed to determine the total volume excreted.

For experiments capturing in situ fascicle penetration in the BITES, the blood-feeding protocol was followed as described above until a mosquito was seen to be engaged and acquiring a blood meal. With a mosquito stably engaged with the BITES, liquid nitrogen was poured over the mosquito–BITES to freeze the stylet mouthparts inside the scaffold. The BITES with the mosquito with the inserted stylet was then carefully placed in a petri dish and fixed immediately with 4% PFA, followed by permeabilization and staining (Section 2.7). The sample was imaged (Section 2.8) immediately to prevent the stylet mouthparts from exiting the BITES.

For experiments involving post-blood-meal (PBM) culturing, the BITES was presented to mosquitoes (*n* = 50) as described. The protocol was modified by covering the BITES with a thin, stretched layer of Parafilm^TM^ during the entire time (20 min) that the BITES was presented to mosquitoes. After the mosquito blood meal acquisition period, the BITES was retrieved and incubated for either 0 min, 20 min, or 3 days in cell culture media containing 1% penicillin–streptomycin for post-blood-meal (PBM) imaging. After the incubation period, the BITES was washed with PBS+ to then be fixed, permeabilized, and stained (Section 2.7 and Section 2.8). 

### 2.6. Fixation, Staining, and Preparation of BITES Constructs

To acquire images before mosquito blood-feeding, the 4-week culture of cellularized Capgel (BITES) was washed 3× with phosphate-buffered saline (PBS+, with calcium and magnesium, ThermoFisher, Waltham, MA, USA). Fixation was performed by incubating the Capgel with 4% paraformaldehyde (PFA, Sigma-Aldrich, St. Louis, MO, USA) for 30 min at room temperature. Next, the BITES was permeabilized with 0.2% Triton X-100 (Sigma-Aldrich, St. Louis, MO, USA) for 15 min, followed by staining the nuclei and actin proteins using NucBlue Live and ActinGreen 488 (ReadyProbe, Invitrogen; Carlsbad, CA, USA), respectively, solvated in PBS+ for 2 h. After washing 3× and submerging in PBS+, the BITES was kept at 4 °C until imaged.

### 2.7. Confocal Microscopy and Stereomicroscopy

Confocal fluorescence imaging was performed on the Capgel–BITES by transferring them to a glass-bottom petri dish (Fluorodish^TM^- Sigma-Aldrich, St. Louis, MO, USA) with a plastic spatula. A Zeiss 710 laser scanning confocal microscope at 20× and 40× magnification was used to acquire images with the Zen 2010 software (Zeiss; Jena, Germany). The samples were excited with a 405 nm wavelength for NucBlue and 488 nm for ActinGreen 488 and z-stacks were acquired in both channels. Differential interference contrast (DIC) imaging was also performed to view cells in the context of the Capgel–BITES capillary structure. The images were overlaid, and maximum-intensity z-projections, 3D projections, and orthogonal views were processed using ImageJ. DIC movies/videos were captured using confocal microscopy and processed with the Zen 2010 software. Images of mosquito stylet mouthparts inside BITES were taken with a stereo microscope equipped with a digital camera (MU1803, AmScope, Irvine, CA, USA). 

### 2.8. Evaluating Nuclear Orientation in BITES Microvessels

To determine the orientation of HDF (*n* = 12 images) and HUVEC (*n* = 6 images) nuclei in capillaries, threshold application was performed on the NucBlue channel maximum projections of z-stacked images taken at cellularized regions perpendicular to the capillaries. These images were binarized in ImageJ and then processed using the “region props” function in MATLAB (vR2021b, MathWorks, Natick, MA, USA). Major and minor axes of the nuclei were used to approximate the elliptical blobs. When the major axes of nuclei were parallel with respect to the image vertical axis by ±20°, nuclear orientation was considered to be aligned with the capillaries. Nuclei with centroids above the image horizontal midline were considered to have orientation values of −90° to 90°, and nuclei with centroids below the midline were considered to have orientations from 90° to 270°.

### 2.9. Statistical Analysis

The median and mean of the number of mosquitoes attempting to feed on the BITES were calculated in Origin 2021 (OriginLab Corporation, Northampton, MA, USA) by analyzing the data in a box chart. Tukey’s outlier detection criterion was applied to determine outliers in the data, using the interquartile range (IQR). Values were labeled as outliers if found to be less than

*minimum = Q*1 − 1.5*IQR* (5)

or if greater than

*maximum = Q*3 + 1.5*IQR* (6)

where *Q*1 is a value that is less than 75% of the points in the dataset, *Q*3 is a value that is greater than 75% of the points in the dataset, and the difference in these values is used to find

*IQR = Q*3 − *Q*1. (7)

Using the Chi-squared (χ^2^) test statistic with a 95% confidence interval and two (2) degrees of freedom, the Marascuillo procedure was used as detailed in the NIST e-Handbook of Statistical Methods to determine the statistical significance of percent mosquito blood-feeding at different BITES presentation times [33].

## 3. Results

**Figure 1:**
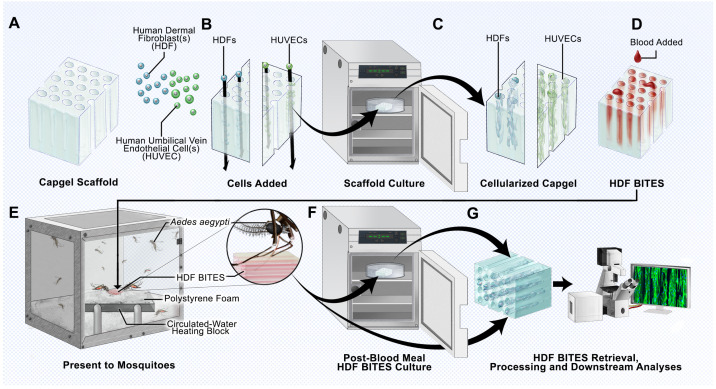
Schematic overview of the BITES platform. (**A**) Illustration of a sterile Capgel block depicting the patent capillary microstructure. (**B**) Loading/charging of the Capgel with either human dermal fibroblasts (HDFs) or human umbilical vein endothelial cells (HUVECs) into the capillaries of the block. (**C**) Culture of the human cell-laden Capgel to cellularize the block. (**D**) Loading of blood into the capillaries of the cellularized Capgel to create a BITES construct. (**E**) Presentation of the BITES construct to female *Aedes (Ae.) aegypti* mosquitoes (prototypic arthropod) for natural biting, probing, and blood-feeding. Inset**:** Zoomed-in depiction of a mosquito with the stylet inserted into the BITES construct, engaging in natural biting/probing/blood-feeding. (**F**) Transfer of the BITES construct from the mosquito cage into an incubator for (optional) post-biting/probing/blood-feeding cell culture. (**G**) Retrieval of the BITES construct following post-biting/probing/blood-feeding cell culture (if any) for processing and various downstream biological analyses.

The outcomes for a host bitten by an arthropod are significantly influenced by the early events occurring at the bite site [9]. We therefore developed the BITES platform using capillary alginate gel scaffolds, human cells, and blood (Figure 1) as a new tool to investigate mosquito biting and blood-feeding.

The 3D microstructures of uniaxial capillaries running in parallel make Capgel an ideal scaffold to template the growth of cultured human cells into hollow tubular microvessel-like structures that can be filled with blood to form a BITES construct (Figure 1A–D). The hydrogel nature of Capgel supports the gentle heating of the construct to a physiological temperature range, i.e., 34–37 °C, and is posited to allow mosquitoes to naturally bite/penetrate, probe for, and blood-feed from the stacked array of blood-filled BITES microvessel structures (Figure 1E and inset). Further, the BITES constructs can then be recovered following arthropod biting/probing/blood-feeding and transferred into media to incubate for additional time in cell culture if desired (Figure 1F). Ultimately, these BITES constructs are to be retrieved, processed, and analyzed, which here included recovery, fixation, staining, and imaging/microscopy (Figure 1G). Herein, BITES was tested against *Ae. aegypti* female mosquitoes as a prototypic biting vector arthropod. 

### 3.1. Blood Meal Acquisition by Ae. aegypti from Blood-Loaded, Non-Cellularized Capgel

**Figure 2:**
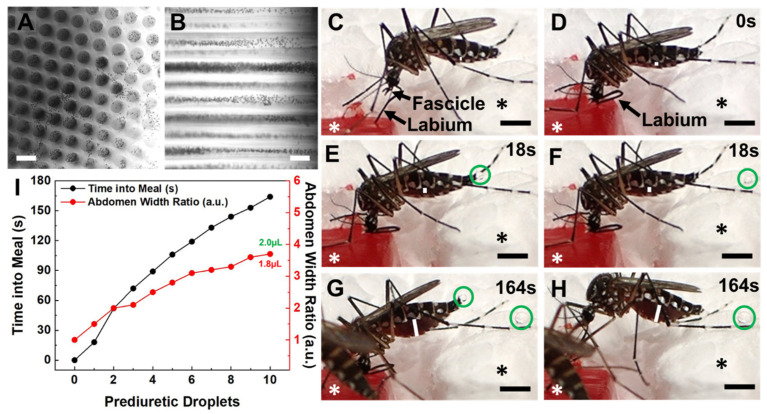
Female *Ae. aegypti* mosquitoes bite into, probe and blood-feed from Capgel loaded with blood in a manner matching the natural taking of a blood meal from a vertebrate host. (**A**) Representative differential interference contrast (DIC) micrograph of a blood-loaded Capgel showing the end-on view of the capillary microstructure, termed *capview*; RBCs within the capillaries and those that have flowed out are observable as multiple small, dark, circular/spherical particles throughout the image. (**B**) Representative DIC micrograph of a blood-loaded Capgel (similar to that of (**A**)) with the long-axis of the capillary in the plane of the image, which is termed *raftview*; RBCs are now observable as multiple small, dark, circular/spherical particles confined exclusively within capillaries. (**C**–**H**) Nonconsecutive, sequential set of image stills taken from video recordings (Videos S3 and S4) capturing the same representative taking of a blood meal event by an *Ae. aegypti* female from a warmed, blood-loaded Capgel block oriented in raftview: (**C**) biting and probing; (**D**) initiation of blood ingestion; (**E**–**H**) feeding to repletion, including prediuretic droplet excretion. (**I**) Plot of abdominal width ratio (Equation (1)) and the number of excreted prediuretic droplets over time of the blood meal taken by a female *Ae. aegypti* mosquito from warmed, blood-loaded Capgel. White asterisks label the warmed, blood-loaded Capgel; black asterisks label the polystyrene foam insulation surrounding the Capgel block. White lines mark the abdominal width of the mosquito and green circles enclose prediuretic droplet(s). The green number is the estimated total volume (including prediuretic excretions) of blood ingested, calculated by approximating the abdomen initial and final volumes as ellipsoids (Equation (2)), taking the difference (Equation (3)), and adding the droplet volumes approximated as spheres (Equation (4)); the red number is the estimated final volume of blood in the midgut only. Scale bars = 50 µm for (**A**,**B**) and 1 mm for (**C**–**H**).

To examine whether the Capgel biomaterials are robust 3D tissue scaffolds for BITES constructs, the loading, distribution, and movement of blood within the Capgel blocks was evaluated, as well as the ability of *Ae. aegypti* females to bite into, probe, and blood-feed from warmed, blood-loaded Capgel (Figure 2). The addition of defibrinated blood to the open ends of Capgel capillaries (termed capview, Figure 2A) rapidly loaded blood into these capillaries and allowed for the visualization of the blood, perpendicular to the capillary long axis, termed raftview (Figure 2B). This blood was confined to and distributed within the capillaries, did not appear to bind to the Capgel walls (specifically the red blood cells—RBCs), and was able to freely move/flow within these microstructures (Video S1), observations that match the expectations of microvessels. Further, when fluid was applied to one end of the capillary, RBCs were observed flowing out of the opposite capillary ends (Video S2). 

The taking of a blood meal by a representative female *Ae. aegypti* mosquito from a warmed (34–37 °C), blood-loaded Cagpel oriented in raftview is summarized in Figure 2, panels C–I (see also Figure S2; Videos S3 and S4). This process/event encompasses the following: biting and probing (Figure 2C); the initiation of blood ingestion (Figure 2D); feeding to repletion, which included prediuretic droplet excretion/expulsion (Figure 2E–H) and took a total of approximately three (3) minutes to complete. As blood ingestion proceeded and the midgut filled, the width of the abdomen correspondingly increased (Figure 2C–H, white lines). 

The relative amount of this steady increase was quantified using an abdomen width ratio metric (Equation (1)) and plotted against the blood-feeding time (Figure 2I, black dots and line). The mosquito began excreting prediuretic fluid in the form of a clear droplet from its anus 18 s after the onset of blood ingestion (Figure 2E, green circle) and forcibly expelled it across a short distance less than a second later (Figure 2F, green circle). The female excreted/expelled nine such additional prediuretic droplets (Figure 2G,H, green circles; Videos S3 and S4) at roughly regular intervals over the remaining 146 s of blood-feeding (Figure 2I, red dots and line; Videos S3 and S4). Approximating a droplet volume as a sphere (Equation (4)) and adding these to the calculated estimate for the final volume of the blood in the midgut yielded 2.0 µL for the estimated total volume of blood ingested by the mosquito (Figure 2I, green number). 

### 3.2. Cellularization of Capgel with HDFs or HUVECs

**Figure 3:**
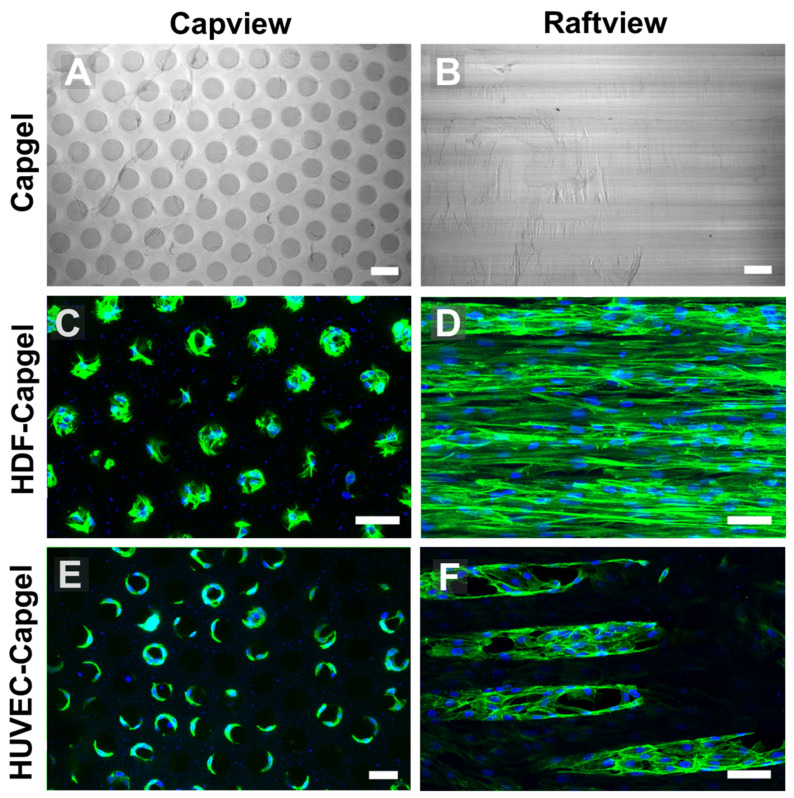
HDFs and HUVECs line scaffold capillaries with tissue when cultured within Capgel biomaterials for four (4) weeks. (**A**,**B**) Representative DIC micrographs of Capgel imaged in capview and raftview orientations, respectively. (**C**,**D**) Representative maximum z-projection confocal fluorescence micrographs of HDF tissue structures formed within Capgel scaffold cultures imaged in capview and raftview orientations, respectively. (**E**,**F**) Representative maximum z-projection confocal fluorescence micrographs of HUVEC tissue structures formed within Capgel scaffold cultures imaged in capview and raftview orientations, respectively. Green fluorescence: Actin Green 488 nm cytoskeletal staining; blue fluorescence: Nucblue nuclei staining. Scale bars = 100 µm for (**A**,**B**) and 50 µm for (**C**–**F**).

The uniform capillary microarchitectures of Cagpel scaffolds were cellularized with either HDFs or HUVECs and then characterized (Figure 3). The Capgel scaffold blocks for cellularization had evenly distributed patent capillaries that were ~50–70 µm in diameter (Figure 3A) and ran parallel to each other from end to end (Figure 3B). Both cell types colonized the Capgel scaffolds during the four-week culture period, especially the HDFs (Figure 3C–F). Cells attached and spread on the Capgel, ultimately lining the capillary walls with tissue (Figure 3C,E). After a month in the scaffold culture, HDFs lined most capillaries with continuous, lengthy (>300 µm) tissue structures (Figure 3D, Video S5). HUVECs also lined multiple Capgel capillaries during this same time in culture with shorter (<200 µm), patchy segments of tissue (Figure 3F, Video S6). 

**Figure 4:**
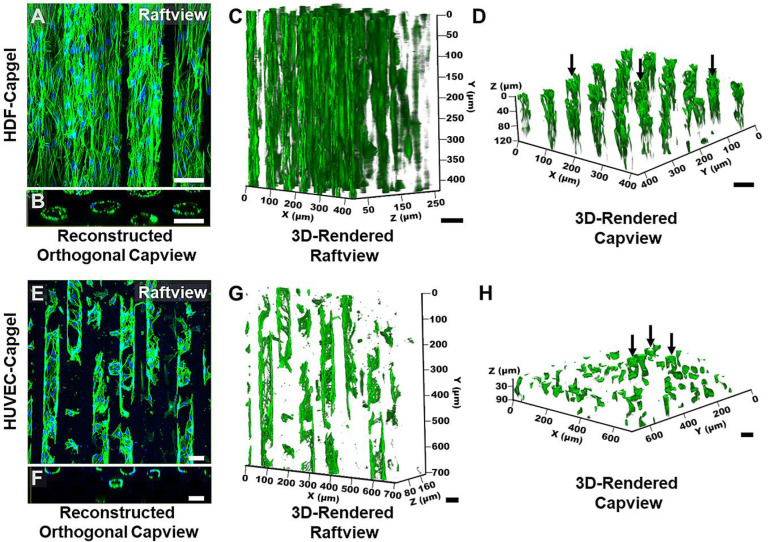
HDFs and HUVECs form stable, tubular 3D tissue structures morphologically similar to microvessels when cultured for four (4) weeks within Capgel biomaterial scaffolds. (**A**,**B**) Representative raftview maximum z-projection confocal fluorescence micrograph and corresponding reconstructed orthogonal capview image, respectively, of tubular HDF tissue structures formed within Capgel scaffolds. (**C**,**D**) Representative raft- and capview snapshots, respectively, from confocal fluorescence microscopy 3D renderings of tubular HDF tissue formations within Capgel. (**E**,**F**) Representative raftview maximum z-projection confocal fluorescence micrograph and corresponding reconstructed orthogonal capview image, respectively, of tubular HUVEC tissue structures formed within Capgel scaffolds. (**G**,**H**) Representative raft- and capview snapshots, respectively, from confocal fluorescence microscopy 3D renderings of tubular HUVEC tissue formations within Capgel. Green fluorescence: Actin Green 488 nm cytoskeletal staining; blue fluorescence: NucBlue nuclei staining. Black arrows indicate fully formed tubular vessels. Scale bars = 50 µm.

The microvessel-like 3D tissue morphologies formed by the cells during culture in the scaffolds are evident in Figure 4. Consistent with the Figure 3 data, the raftview maximum z-projection confocal fluorescent micrographs show the extensive HDF tissue formations throughout the Capgel (Figure 4A) and the focal structures formed by HUVECs (Figure 4E). Also consistent are the associated reconstructed orthogonal capviews showing that both HDFs and HUVECs lined the capillary walls to form patent, tubular tissues (Figure 4B,F, respectively). The dense scaffold cellularization by HDFs is evident in the 3D raft- and capview renderings (Figure 4C,D, respectively), as is the more diffuse HUVEC cellularization of the Capgel (Figure 4G,H, respectively).

**Figure 5:**
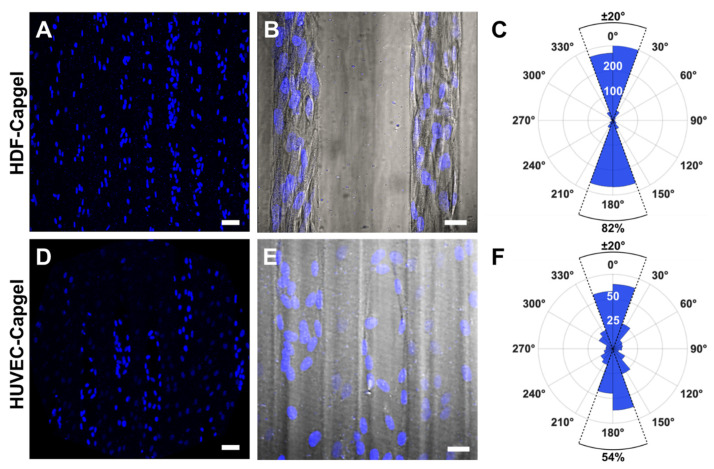
HDFs and HUVECs comprising microvessel tissues engineered with Capgel biomaterials align preferentially in the direction of the scaffold capillary long axis during four (4) weeks in culture. (**A**) Representative confocal fluorescence maximum z-projection micrograph showing NucBlue-stained nuclei of HDFs cultured in Capgel. (**B**) Overlay image of representative confocal fluorescence maximum z-projection and corresponding DIC micrographs of scaffold-cultured HDFs stained with NucBlue. (**C**) Polar plot quantifying the nuclear alignment of HDFs cultured in Capgel as determined by first fitting ellipses to binarized images (*n* = 12) of NucBlue-stained nuclei and then measuring the angle of each best-fit ellipse major axis relative to the scaffold capillary long axis defined as either 0° or 180°. The upper half of the polar plot ranging from −90° to 90°, which includes 0°, describes nuclei in the top half of the analyzed images, while the lower half of the plot from 90° to 270°, which includes 180°, describes the nuclei in the bottom half of these images. The radial value of the arcs for blue-shaded sectors indicates the total number of nuclei, i.e., cells, within the covered range of relative angles. (**D**–**F**) These panels are the same types of images and plots as panels (**A**–**C**), respectively, but for HUVECs scaffold-cultured in Capgel (*n* = 6 for (**F**)). Blue fluorescence: NucBlue nuclei staining; gray: DIC. Scale bars = 50 µm for (**A**,**D**), 20 µm (**B**,**E**).

The engineered microvessel tissues adopted orientations parallel to the long axes of scaffold capillaries (Figure 5). Confocal fluorescence maximum z-projection micrographs of HDFs and HUVECs stained with NucBlue clearly show elliptically shaped nuclei preferentially elongated in the capillary long-axis direction, i.e., vertically in the images (Figure 5A,D, respectively). Fluorescent micrographs captured at an increased magnification and overlaid with the associated DIC images show that HDF and HUVEC cell bodies were also elongated preferentially in this same direction (Figure 5B,E, respectively). Quantification of cell orientations in the scaffold capillaries revealed that 84% of HDFs and 54% of HUVECs were oriented within ±20° of the capillary direction (Figure 5C,F, respectively).

### 3.3. Blood Meal Acquisition by Ae. aegypti from BITES Tissue

**Figure 6:**
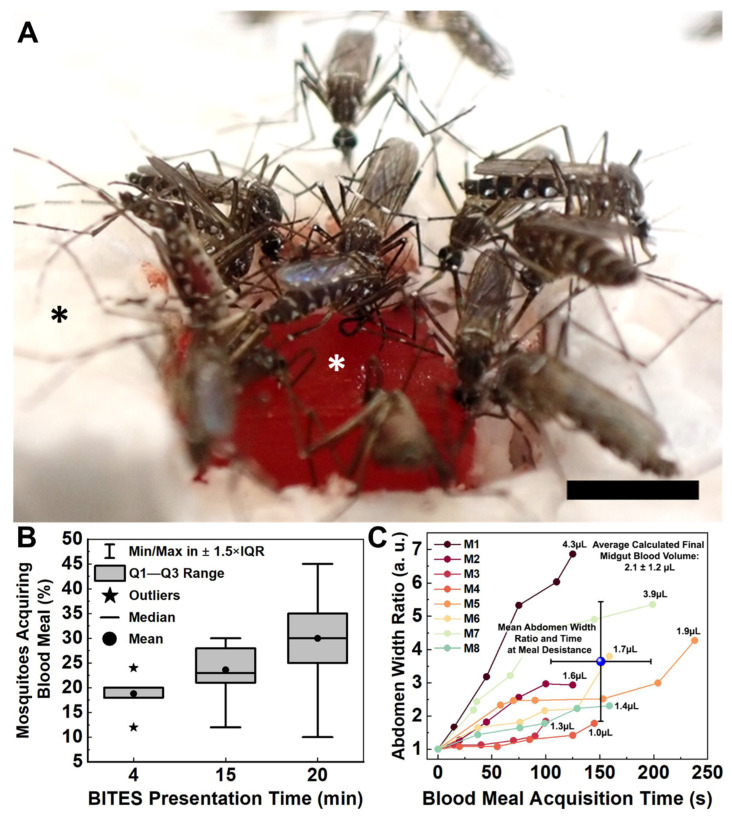
Female *Aedes aegypti* mosquitoes swarm warmed BITES tissue loaded with blood and engage in frenzied blood meal acquisition behaviors. (**A**) Representative image captured from a video (Video S9) presenting a warmed (34–37 °C), blood-loaded BITES construct (cellularized with HDFs) in the raftview orientation to 50 mosquitoes for 15 min. (**B**) Box plot summarizing the percentages of *Ae. aegypti* (out of 50 total) that demonstrated blood meal acquisition behaviors on BITES human dermal microvascular bed model tissues (HDF-cellularized) presented for either 4, 15, or 20 min in separate experiments. (**C**) Plot of abdomen width ratio (Equation (1)) increases over acquisition time for six (6) individual females taking blood meals from an HDF BITES construct presented for 15 min (Video S9). The average abdomen width ratio at the average time to blood meal desistance for all mosquitoes analyzed is indicated by the blue sphere in the plot, with error bars for both values. Numbers listed next to each final width ratio point indicate the final estimates of blood volumes in the corresponding mosquito midguts, calculated as before (Equations (2) and (3)). In panel (**A**), the white asterisk indicates the HDF-cellularized BITES tissue loaded with blood; the black asterisk indicates polystyrene foam insulation. In the box plot (**B**), vertical brackets = whiskers within Tukey’s 1.5 × interquartile (IQR) range, gray boxes = IQR, stars = outliers, horizontal lines = medians, dots = means; note that the median for 4 min equals/overlaps Q3 and the whiskers for this presentation time fall within the IQR and therefore are not shown. Error bars in (**C**) = ± standard deviation (SD). Scale bar = 2 mm.

Blood was easily loaded into BITES tissue (Figure 6A, white asterisk, raftview orientation) and not restricted by the HDF cellularization. Mosquitoes swarmed the BITES warmed (34–37 °C) constructs, frequently biting, probing, and/or blood-feeding (Figure 6A, Video S9).

Warmed BITES constructs were presented to 50 Female *Ae. aegypti* for 4, 15, or 20 min in independent experiments (Figure 6B, Videos S9–S11, respectively). The mean percentages (±standard deviation-SD) and medians of mosquitoes engaging in blood meal acquisition behaviors on the BITES tissue (i.e., biting, probing, and/or blood-feeding) were 19 ± 4.4%, 20%; 24 ± 4.9%, 23%; and 30 ± 8.2%, 30% for the 4, 15, and 20 min presentations, respectively (Figure 6B, dots for means, horizontal lines for medians). The interquartile ranges (IQR) increased from 2% for the 4 min presentation to 6.5% for the 15 min presentation and to 10% for the 20 min presentation (Figure 6B, gray boxes). Outliers—defined as those values outside of Tukey’s range—were only observed for the 4 min presentation (Figure 6B, stars, 12% and 24%). All other values observed for the different BITES presentation times fell within the whisker ranges of the box plot (Figure 6B, vertical brackets). Using the Marascuillo procedure [33], no significant differences were found for any of the mean proportions of females engaging BITES during the different presentation times. 

Individual *Ae. aegypti* had a range of engorgement levels after taking blood meals from the BITES tissue (Figure 6C). In less than a minute, five of the six mosquitoes analyzed (10% of the total mosquitoes in the cage) had visually detectable midgut distention with abdomen width ratios (Equation (1)) greater than 1.5. The average time to blood “meal desistance” [34] was consistent between the females, with a mean ± SD of 151 ± 46 s (Figure 6C, blue sphere, horizontal error bars). The average level of engorgement (i.e., abdomen width ratio) at this average desistance time was more varied, having a mean ± SD of 3.6 ± 1.8, and the average calculated estimate for the final blood volume in a mosquito midgut (Equations (2) and (3)) was 2.1 ± 1.2 µL (Figure 6C, blue sphere, vertical error bars; upper right corner text).

### 3.4. In Situ Characterization of Ae. aegypti Stylet Mouthparts in BITES Microvascular Beds and Post-Blood-Meal BITES Culture

**Figure 7:**
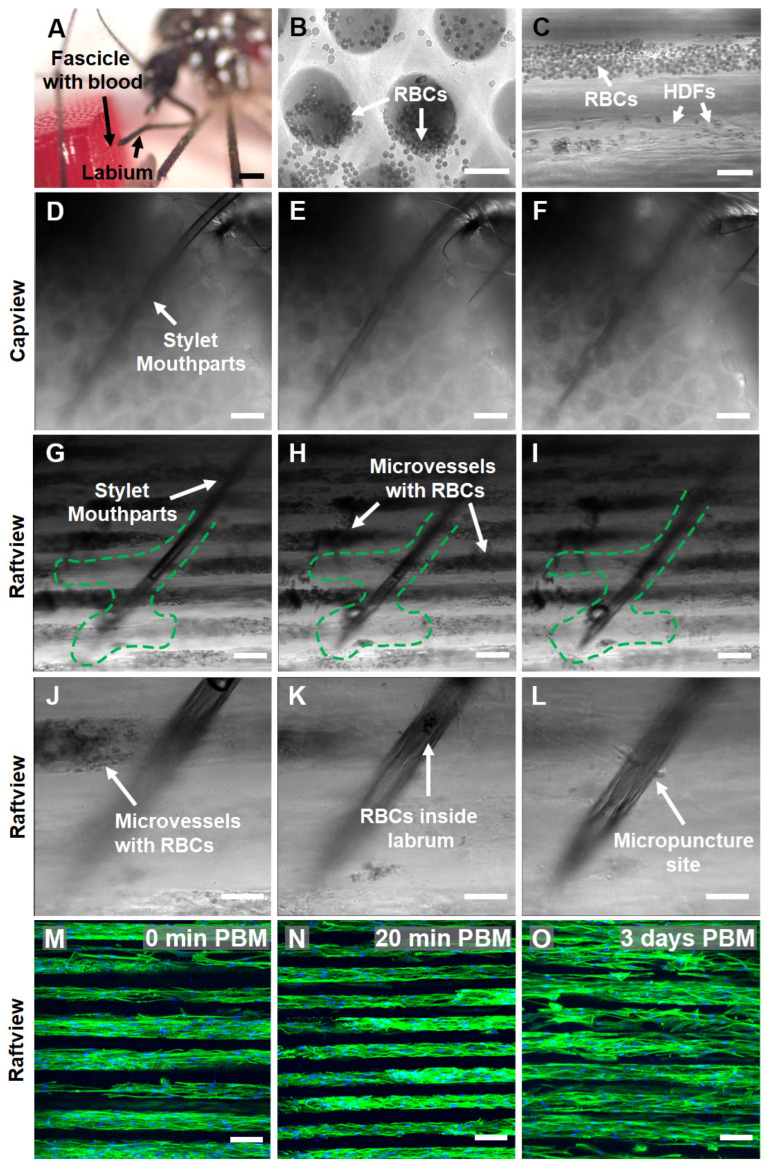
Female *Ae. aegypti* penetrate through multiple layers of warmed, blood-loaded HDF BITES microvessel tissue structures with the fascicle of stylet mouthparts to take a blood meal and the tissues can be cultured for days post-blood-meal (PBM). (**A**) A stereomicrograph of a mosquito snap-frozen with liquid nitrogen during blood meal acquisition from an HDF BITES tissue loaded with blood. The fascicle filled with blood can be seen penetrating the right side of the tissue in the raftview orientation, with the labium characteristically bent and pulled away. (**B**,**C**) Representative DIC micrographs of HDF BITES microvessels filled with blood imaged in the raftview and capview orientations, respectively. RBCs confined within microvessels and that have flowed out of the ends are observable as multiple small, dark, circular/spherical particles in the images (see also Videos S7 and S8). (**D**–**I**) Representative series of DIC micrographs at different focal planes showing the mosquito fascicle/stylet mouthparts penetrating HDF BITES tissue (**A**) imaged in in situ, capview, and raftview orientation, respectively. (**J**–**L**) Representative DIC micrographs at different focal planes and increased magnification relative to (**G**–**I**), showing a zoomed-in view of the end of the stylet mouthparts within the HDF BITES tissue (see also Video S13). (**M**–**O**) Representative confocal fluorescence maximum z-projection micrographs of HDF BITES tissues imaged in the raftview orientation immediately following presentation and blood meal acquisition by *Ae. aegypti* (0 min PBM), or after additional short (20 min) or longer (3 days) PBM culture times (see also Video S15). Green dashed lines outline “interaction volumes” of the stylet mouthparts within HDF BITES tissue. Green fluorescence: Actin Green 488 nm cytoskeletal staining; blue fluorescence: Nucblue nuclei staining; gray: DIC. Scale bars = 1 mm for (**A**), 50 µm for (**B**,**C**,**J**–**L**), and 100 µm (**D**–**I**,**M**–**O**).

To assess the microscopic interactions of *Ae. aegypti* fascicles of stylet mouthparts penetrated BITES microvessel beds, mosquitoes were first snap-frozen with liquid nitrogen mid-blood-meal acquisition from BITES tissue cellularized with HDFs (e.g., end of Video S10). Snap-freezing captured the penetration of the mosquito fascicle/stylet mouthparts into a blood-loaded BITES tissue and fixed it in situ (Figure 7A). 

DIC micrographs and corresponding supplementary videos show that blood was loaded and able to freely move/flow within the microvessel structures of HDF-cellularized BITES tissue (Figure 7B,C, Videos S7 and S8). A series of DIC micrographs at different focal planes of the situation, pictured in Figure 7A, revealed that the *Ae. aegypti* pushed the stylet mouthparts through/past multiple layers of blood-filled BITES microvessel structures to acquire the blood meal (Figure 7D–F, Video S12, capview orientation; Figure 7G–I, raftview and Video S13).

It is conspicuous that areas near the fascicle track and at the end of the stylet mouthparts in the raftview images were devoid of RBCs (Figure 7G–I, green dashed lines). A 3D-rendered depth profile created by leveraging the autofluorescence of the fascicle in the 488 nm channel indicated that the penetration depth of the stylet mouthparts into the BITES tissue was ~500 µm (Figure S3B). Another series of DIC micrographs zoomed in to the end of the fascicle show this “interaction volume” in greater detail (Figure 7J–L, Video S14), including RBCs inside the labrum and the micropuncture site of a BITES microvessel structure by the stylet mouthparts (Figure 7J,L, respectively).

To test whether the BITES tissues could be cultured following blood meal acquisitions by *Ae. aegypti* females, HDF-cellularized BITES tissue was first covered with a thin Parafilm^TM^ membrane and then presented to mosquitoes. Maximum z-projection confocal fluorescence micrographs of these BITES microvessel bed tissues showed that the HDFs and microvessel structures were intact immediately (0 min) post-blood-meal (PBM) as well as after short (20 min) and longer (3 days) times in culture PBM (Figure 7M–O, respectively; Video S15). Notably, no obvious signs of contamination, such as yellow cloudy media, aggregates of bacteria, or fungal hyphae, were observed in the 3-day PBM BITES tissue culture.

## 4. Discussion

The implementation of bioengineering and tissue engineering approaches to the study of vector arthropod control and bite-site biology is a nascent area of investigation [22,24,35,36]. The great promise of this developing area is clear and includes engineered avascular human skin equivalents [24], silicone microfluidic chambers for the direct collection and high-throughput downstream analysis of mosquito saliva [36], and acellular 3D-printed model skin composed of poly(ethylene glycol) diacrylate (PEGDA) and gelatin methacrylate (GelMA) biomaterials that include circulating blood for mosquito feeding [22]. Detailed here are the initial proof-of-concept studies of 3D human dermal microvessel bed tissue models engineered with Capgel biomaterial scaffolds that female *Ae. aegypti* mosquitoes bit, probed, and blood-fed from naturally. We termed this new in vitro platform for the study of arthropod bite-site biology a Biologic Interfacial Tissue-Engineered System (BITES, Figure 1).

Blood meal acquisition by *Ae. aegypti* from warmed (34–37 °C), blood-loaded Capgel was documented with videography and examined. The defibrinated bovine blood was easily loaded into the Capgel and capable of freely flowing in the scaffold capillaries (Figure 2A,B, Videos S1 and S2), which is required for uptake by the mosquito. In nature, mosquitoes generally engage in foraging behavior when seeking host blood meals [37]. After a host is found, *Ae. aegypti* initiate the blood-feeding process by first biting/penetrating the skin with the fascicle containing the stylet mouthparts and then probing the dermis for a blood microvessel(s) [38,39]. Once a suitable microvessel(s) is identified, the stylet mouthparts are fully inserted and the labium (i.e., sheath) part of the proboscis pulls away from the stylet and remains outside the host skin [39,40]. All these behaviors were clearly demonstrated by female *Ae. aegypti* during blood meal acquisition from the blood-loaded, warmed Capgel in this study (Figure 2C,D and Figure S2A,B, Video S3 and S4).

A mosquito starts expectorating saliva from the hypopharynx stylet mouthpart as soon as the skin is penetrated by the fascicle to act as a lubricant, inhibit coagulation, and modulate host immune responses [40,41,42]. The female then begins to ingest blood through a combined structure similar to a straw, formed by the hypopharynx and labrum stylet mouthparts [38]. This blood then fills the midgut, which is accompanied by an increase in abdominal width [40]. Though the expectoration of saliva was not directly observed from mosquitoes engaging in blood meal acquisition behaviors from Capgel, blood filling the midgut and associated abdominal expansion was seen, and these processes—saliva expectoration and blood ingestion—are inextricably linked biologically (Figure 2D–I and Figure 2Figure 2 S2C–U, Videos S9–S11).

In a given meal, a mosquito generally ingests more weight in blood than its total body weight [43], and there is a possibility of midgut rupture if more than ~5–7 µL of blood is ingested [40]. To avoid rupture, mosquitoes engage in a prediuretic process to concomitantly excrete 10 or more droplets of urine during an average blood meal, to concentrate the erythrocyte content (nutrients) in the midgut and effectively increase the total protein intake [44,45]. Additionally, prediuresis cools the mosquito and keeps the body temperature within safe physiological limits [46]. Blood-feeding times for *Ae. aegypti* range from 32.7 to 307.6 s according to Ribeiro et al. [34], and Stobbart reports 2.50 ± 0.66 µL for the mean (±SD) repletion volume (not corrected for prediuresis) [47]. In this study, the *Ae. aegypti* spent 163 s taking a blood meal from the warmed, blood-loaded Capgel, excreted 10 prediuretic drops during that time, had a final abdomen width ratio of 3.9, and ingested a calculated estimate of 2.0 µL of blood in total, i.e., including the estimated 0.2 µL of prediuretic excretions (Figure 2 and Figure S2 and Videos S3 and S4). Altogether, these results strongly support the assertion that female *Ae. aegypti* mosquitoes acquired blood meals from warmed, blood-loaded Capgel in a manner mimicking the typical natural behaviors exhibited during the taking of a blood meal from vertebrate hosts.

The unique uniform capillary microstructure of Capgel biomaterials provides an excellent tissue scaffold to engineer stylized dermal microvessel beds (Figure 3A,B) with capillary channel diameters between ~50 and 70 µm, approximating those of the dermal microvasculature once cellularized [48]. Fibroblasts and endothelial cells (here, specifically HDFs and HUVECs) were chosen for these proof-of-concept experiments because both cell types are integral components of microvessels and the dermis is populated in part by fibroblasts [49,50,51,52,53]. Further, emphasis was placed on modeling the dermis/dermal microvessels because this tissue is the primary target for the bites of hematophagous arthropods such as mosquitoes. 

Both HDFs and HUVECs colonized the Capgel scaffolds and lined the capillary channels with patent, 3D tubular, microvessel-like tissues composed of oriented cells (Figure 3C–F, Figure 4 and Figure 5, Videos S5 and S6). The HDF microvessel structures were longer and contiguous within the scaffolds, compared to the patchy HUVEC microvessel tissues (Figure 4, Videos S5 and S6). In natural vessels, cells are preferentially orientated in the direction of blood flow, and this alignment reflects and facilitates the structure and function [54,55,56,57]. Analogously, the majority—82% and 54% of HDFs and HUVECs, respectively—of cells cultured in Capgel were also aligned (±20°) in the direction of the scaffold capillary long axis (Figure 5). These alignment behaviors likely stem from both the inherent capacities of the cells to orient in response to scaffold features and the configuration of the Capgel scaffolds. Similar alignment phenomena have been documented previously for fibroblasts and endothelial cells (60% and 50%, respectively) cultured in confined, 50-µm-wide, gelatin methacrylate hydrogels [58]. Overall, these results suggest that the scaffold culture conditions were sufficient for HDFs but may need to be adjusted for HUVECs. Such adjustments could include (1) an increased initial HUVEC seeding density, (2) longer culture periods, and/or (3) co-culture in scaffolds first lined with HDF structures. The co-culture approach is particularly attractive and will be a focus of future BITES work as it is known that these cell types cooperate to form robust tubular endothelial structures and this arrangement mimics the natural microanatomy [49,50,51,52].

Once the BITES constructs were developed, experiments were conducted to evaluate the acquisition of blood meals by female *Ae. aegypti* mosquitoes from these engineered tissues. Only BITES tissue cellularized with HDFs was advanced into these proof-of-concept experiments because it was the best model representation of fully cellularized beds of microvessel tissue structures engineered with human cells. Mosquitoes swarmed the HDF BITES that was warmed and loaded with blood (Figure 6A, Videos S9–S11), indicating that they were attracted to the tissue. The percentage of *Ae. aegypti* that engaged the engineered tissue was similar for different presentation times (Figure 6B, Videos S9–S11), which suggests that these constructs need only be presented to mosquitoes for tens of minutes at most to conduct effective experiments. On average, an individual female took 151 ± 46 s to acquire a blood meal (roughly in the middle of Ribeiro et al.’s range [34]), and the relative amount of blood ingested quantified by the nondimensionalized abdomen width ratio metric was 3.6 ± 1.8, and the calculated estimate of the blood volume in the midgut was 2.1 ± 1.2 µL (Figure 6C). The largest observed ratio was almost seven (7), which corresponded to 4.3 µL of blood (calculated estimate) in the midgut of this mosquito, and 37.5% of the analyzed *Ae. aegypti* fed near or to repletion, with calculated midgut blood volumes of 1.9 µL or above (i.e., within 0.66 µL—one SD—of 2.5 µL [47]). All these data together strongly support the assessment that blood meal acquisitions by female *Ae. aegypti* mosquitoes from warmed, blood-loaded HDF BITES microvessel tissue beds mimic natural acquisitions from vertebrate hosts.

As with the blood-loaded Capgel, HDF cellularization did not prevent the free movement/flow of blood within the BITES microvessels (Figure 7B,C, Videos S7 and S8). A DIC micrograph and corresponding 3D-rendered depth profiling of the situation revealed that the mosquito pushed the fascicles of stylet mouthparts ~500 µm into the BITES tissue, a depth at which dermal microvessels would be encountered in humans (Figure S3) [48,59]. A closer inspection of this penetration by DIC microscopy in the capview and raftview orientations showed that several layers of the microvessel structures were pierced (Figure 7D–I, respectively) and a track with an irregular “interaction volume” was created (Figure 7G–I, green dashed lines). The paucity of RBCs observed in this track and interaction volume indicates that the total volume of the blood meal was extracted from multiple BITES microvessel structures, even with the apparent narrowness of the punctures (Figure 7J–L). Confocal microscopy additionally demonstrated that the BITES microvessel structures were intact after blood meal acquisitions and that BITES tissue can be viably cultured for at least three days following presentation to and engagement by mosquitoes (Figure 7M–O). No signs of apparent contamination were observed in these post-blood-meal BITES tissue cultures. Antibiotics (penicillin and streptomycin) were standard components of all culture media in this study, helping to reduce/abrogate the bacterial load. The use of these compounds in future BITES studies with other potential vectors (e.g., ticks) and pathogens such as *Borrelia burgdorferi* will likely require careful consideration of both the antibiotic dose and class used, if any. Similar considerations may also be important if microbiome studies are envisioned. These data, especially the demonstration that BITES tissue can be cleanly cultured post-blood-meal for some days, highlight the potential application of the platform in future studies investigating phenomena such as vectored pathogen infection sequences, kinetics, and sequelae.

Disease outcomes for a host bitten by an infected arthropod are closely linked to the early events that occur during the bite, such as host immune modulation and pathogen replication/enhancement at the skin bite site [9]. These events play a crucial role in the development of a disseminated infection within the host and the continuation of the pathogen transmission cycle [6]. The interplay between the biting arthropod, its expectorated saliva, the transmitted pathogens, and the host cells at the skin bite site is essential to understanding these complex interactions [6,60,61,62,63]. Gaining this understanding is vital for the development of effective therapies and preventative strategies against arthropod-borne pathogens and diseases. Numerous studies involving various viruses and mosquito species have shown that, along with the pathogen’s strategies to overcome the host immune response, the expectorated saliva also has a critical role in the initial pathogen replication process [6,60,61,62,63]. However, live animal models often use artificial infection, such as intravenously or intraperitoneally, which fails to accurately replicate natural vector-bite mediated pathogen delivery [64,65]. Even if the pathogens are injected intradermally, this delivery method does not fully replicate delivery by natural biting/blood-feeding [66]. 

Considering these limitations, researchers have turned to bioengineering approaches to study the intersection of mosquito feeding behavior and host skin responses. For example, Janson et al. described acellular 3D-printed “skin” with uniform vasculature architectures as an automated mosquito feeding platform [22]. This platform had large, acellular, vessel-like structures (~750 µm diameter) and was used to evaluate the repellency of DEET and lemon eucalyptus oil as a high-throughput repellent screening platform. This system as described did not incorporate any cell of the skin (resident or migratory) and therefore does not currently offer the potential to investigate vector–host–pathogen dynamics at the cellular or molecular levels. The FTSEs developed by Reuter et al., using type I collagen, HDFs, and human epidermal keratinocytes (hEKs), are a promising development for the study of the biology of the skin bite sites created by arthropods [24]. These authors did identify issues with the mechanical stability of the construct, reporting that the gel contracted by 50% during the first seven (7) days of culture, and the avascular nature of this model precludes its employment in arthropod blood-feeding studies [24]. The BITES platform presented in this study possessed microvessel structures (in contrast to Reuter et al.) and these were approximately an order of magnitude smaller in diameter than those described in the Janson et al. report [20]. The smaller diameters more closely approximate the sizes of microvessels found at depths in the dermis that mosquitoes access for blood-feeding [48,59,67]. Further—distinct from Janson et al.—these BITES microvessel structures resulted from Capgel self-assembly, thus not requiring 3D printing, and were cellularized.

There are some noteworthy limitations of the present study. First, the results here with the BITES platform are necessarily confined to mosquitoes, specifically *Ae. aegypti*. Hence, more studies are needed in the future that explore BITES tissues with other mosquito species, as well as other types of arthropods (e.g., ticks). Second, the BITES model system presented here is an initial platform construct and will need additional cellular elements to more completely approximate human skin. As noted above, our next efforts will focus on the endothelialization of the BITES microvessel structures via HDF–HUVEC co-culture approaches for future investigations with mosquitoes and other hematophagous biting arthropods. Moreover, future studies involving pathogens should incorporate other important skin cells and components (e.g., epidermal keratinocytes and macrophages) into BITES model constructs to more fully investigate responses at the bite site. Further, in this current proof-of-concept iteration of the BITES platform, fibroblasts line the capillary channels and are not diffusely distributed throughout the matrix of the construct [68]. Hence, subsequent iterations of the platform will also leverage 3D bioprinting with Capgel biomaterial inks [31] in conjunction with co-culture approaches to address this limitation. Third, a pumped, directional blood flow was not an incorporated feature into the BITES platform as presented and heat was the singular attractive cue provided. This lack of flow likely impacted mosquito blood meal acquisition and will be required for other types of arthropods, e.g., ticks. Understanding this, the development of a flow cell and system for the BITES platform is currently underway. Defibrinated bovine blood is a commercially available reagent validated and used in our laboratory to feed mosquitoes. In addition, bovine blood products (e.g., serum) are routinely used in media to culture and maintain human cells in vitro. The focus of this study was to demonstrate and characterize mosquito biting and blood-feeding on the BITES platform (i.e., Capgel biomaterials cellularized with human cells). We therefore determined to use defibrinated bovine blood in the system because it is the mosquito blood-feeding standard in our laboratory and was compatible with the cells, which had already been cultured in a fetal-bovine-serum-containing medium. Future studies may need to consider using whole human blood in the BITES platform to improve the model, as fibrinogen/fibrin is a vital component in blood clotting, wound repair, triggering IL-6-related immune reactions, and macrophage adhesion [69]. Such considerations will also likely be especially important for future studies focusing on the responses from mononuclear cell blood components in response to biting/blood-feeding. The addition of volatile attractants to the platform, such as lactic acid, ammonia, and/or CO_2_, is anticipated to be straightforward and would likely improve mosquito/arthropod engagement [70,71]. Finally, future studies will expand into pathogen transmission experiments and the characterization of inflammatory responses using the BITES platform. Our previous publications on Capgel-based engineered tissues demonstrate that standard downstream analytical methods such as in situ live/dead staining, fixation/embedding/sectioning for histological/immunohistochemical assessments, and/or trypsinization to dissociate and isolate cells can be readily applied [25,26,27,28,29,30]. Given this prior experience, we envision that future downstream analyses of BITES tissue could potentially include flow cytometry, the determination of gene and protein expression profiles, and/or timecourse studies of pathogen infection sequences and kinetics.

## 5. Conclusions

In this proof-of-concept study, we first demonstrated that cultured human cells (fibroblasts or endothelial cells) formed stable tubular structures of aligned cells inside Capgel scaffold capillaries into which blood could be freely loaded and move within the lumens. Then, using female *Ae. aegypti* as a prototypic arthropod, we demonstrated that BITES in combination with modest heating (34–37 °C) robustly attracted mosquitoes that bit into, probed, and acquired blood meals from the platform. Further, BITES dermal microvessel tissue bed constructs could be readily cultured for days post-mosquito-biting and blood-feeding. All the promising results of this initial study are the first steps down the fruitful new path of future investigations using the BITES platform to facilitate innovative studies of the skin bite sites of hematophagous vector arthropods.

## 6. Patents

B.J.W. is an inventor on several US and international patents and patent applications. These include US-7,601,525-B2 (Appl. No.: 11/074,285, PCT/US2005/087287) and 63/410,352 and 63/431,361, with the former listing B.J.W. and A.P. as inventors and the latter listing B.J.W., M.D., and M.W.J. as inventors. B.J.W. is an inventor on US-9,258,988-B2 and US-20150020439-A1 (Appl. No.: 14/332,747), as well as EP3021665B1 and EP3021665A1 and EP3021665A4 (Appl. No.: EP14826098A, PCT/US2015/009818). Additionally, B.J.W. is an inventor on patent US-10,948,491-B2 and US-20210239699-A1 and US-20180231550-A1 (Appl. No.: 15/751,638, PCT/US2017/027677), and US-11,020,496-B2 and US-20210283276-A1 and US-US-20190076556-A1 (Appl. No.: 16/127,587). Moreover, B.J.W. is an inventor on US-11,458,042-B2 and US-020180078423-A1 (Appl. No.: 15/568,767), EP-3285783-B1 and EP-3285783-A2 and EP-3285783-A4 (Appl. No.: EP-16824826-A), and CN-107847633-B and CN-107847633-A (Appl. No.: CN-201680036563-A, PCT/US2017/011050A3). The above list is not exhaustive.

## Data Availability

Data are contained and available within this manuscript, supplementary material, and/or from the corresponding author upon request.

## Supplementary Materials

The following supporting information can be downloaded at: https://www.mdpi.com/article/10.3390/insects14060514/s1, Figure S1: BITES Feeding Videography Set-Up; Figure S2: Frames Capturing Prediuresis During BITES Feeding; Figure S3: Stylet Penetration into BITES; Video S1: RBC Flow in Capgel from Raftview Perspective; Video S2: RBC Flow in Capgel from Capview Perspective; Video S3: *Aedes aegypti* Blood-Feeding from Capgel; Video S4: Continuation of Mosquito Feeding on BITES; Video S5: Capillaries Lined with HDF 3D Reconstruction from Capview Perspective; Video S6: Capillaries Lined with HUVEC 3D Reconstruction from Raftview Perspective; Video S7: RBCs Flowing in HDF-BITES from Raftview Perspective; Video S8: RBCs Flowing out of Capillaries of HDF-BITES; Video S9: Video-Recording of Mosquitoes Feeding from BITES (15 min Presentation Time); Video S10: Video-Recording of Mosquitoes Feeding from BITES (4 min Presentation Time); Video S11: Video-Recording of Mosquitoes Feeding from BITES (20 min Presentation Time); Video S12: DIC Images at Different Focal Planes of BITES with Stylet Penetrating from Capview Perspective; Video S13: DIC Images at Different Focal Planes of BITES with Stylet Mouthparts Penetrated from Raftview Perspective; Video S14: DIC Images at Different Focal Planes of Stylet Mouthparts Penetrated BITES with RBCs Visible in the Labrum; Video S15: Merged DIC and Fluorescence 3D Projection of HDF-BITES from Capview Perspective.

## Abbreviations

*Ae.*	*Aedes*
BITES	Biologic Interfacial Tissue-Engineered System
Capgel	Capillary alginate gel
DEET	N,N-diethyl-meta-toluamide
DENV	Dengue virus
DMEM	Dulbecco’s Modified Eagle Medium
DI	Deionized
DIC	Differential interference contrast
FBS	Fetal bovine serum
FTSE	Full-thickness skin equivalents
HDFs	Human dermal fibroblasts
HUVECs	Human umbilical vein endothelial cells
IQR	Interquartile range
LVES	Large vessel endothelial supplement
PBM	Post-blood-meal
PBS	Phosphate-buffered saline
PFA	Paraformaldehyde
RBC	Red blood cell
SEM	Standard error of the mean
SD	Standard deviation
ZIKV	Zika virus
