# Supplementary material for: Engineered Human Tissue as A New Platform for Mosquito Bite-Site Biology Investigations
Source: Insects. 2023 Jun 2;14(6):514. doi: 10.3390/insects14060514 (PMC10299109; doi:10.3390/insects14060514)
Supplement: Supplementary file 1 [file insects-14-00514-s001.zip › Supplemental_Material_Willenberg_BITES_MDPI_Insects_230322.pdf]

Supplementary Information

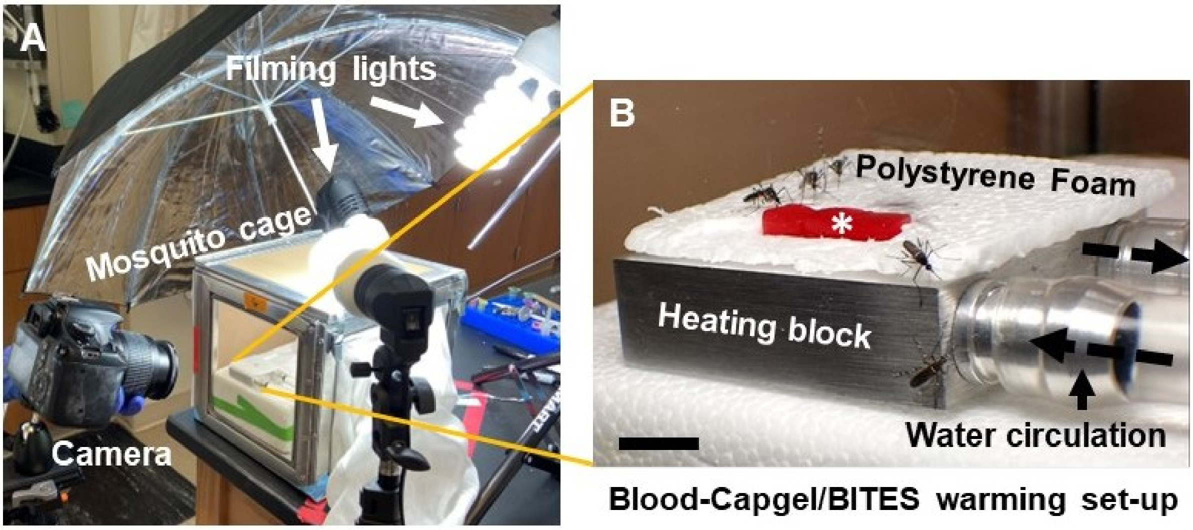

Figure S1: Videography set-up for filming *Ae. aegypti* mosquitoes biting into, probing, and blood-feeding from blood-loaded Capgel or BITES. (**A**) A mosquito cage was used to contain female mosquitoes (n=20-50) and was placed under filming lights/umbrellas with the camera positioned at the optically clear side of the cage to video-record mosquito feeding behavior on BITES. (**B**) BITES (white asterisk) was placed atop a water circulating aluminum heating block to achieve 37°C during mosquito feeding. Bare surfaces of the heating block were covered with polystyrene foam insulation to localize heated area to BITES. Scale bar – 10mm.

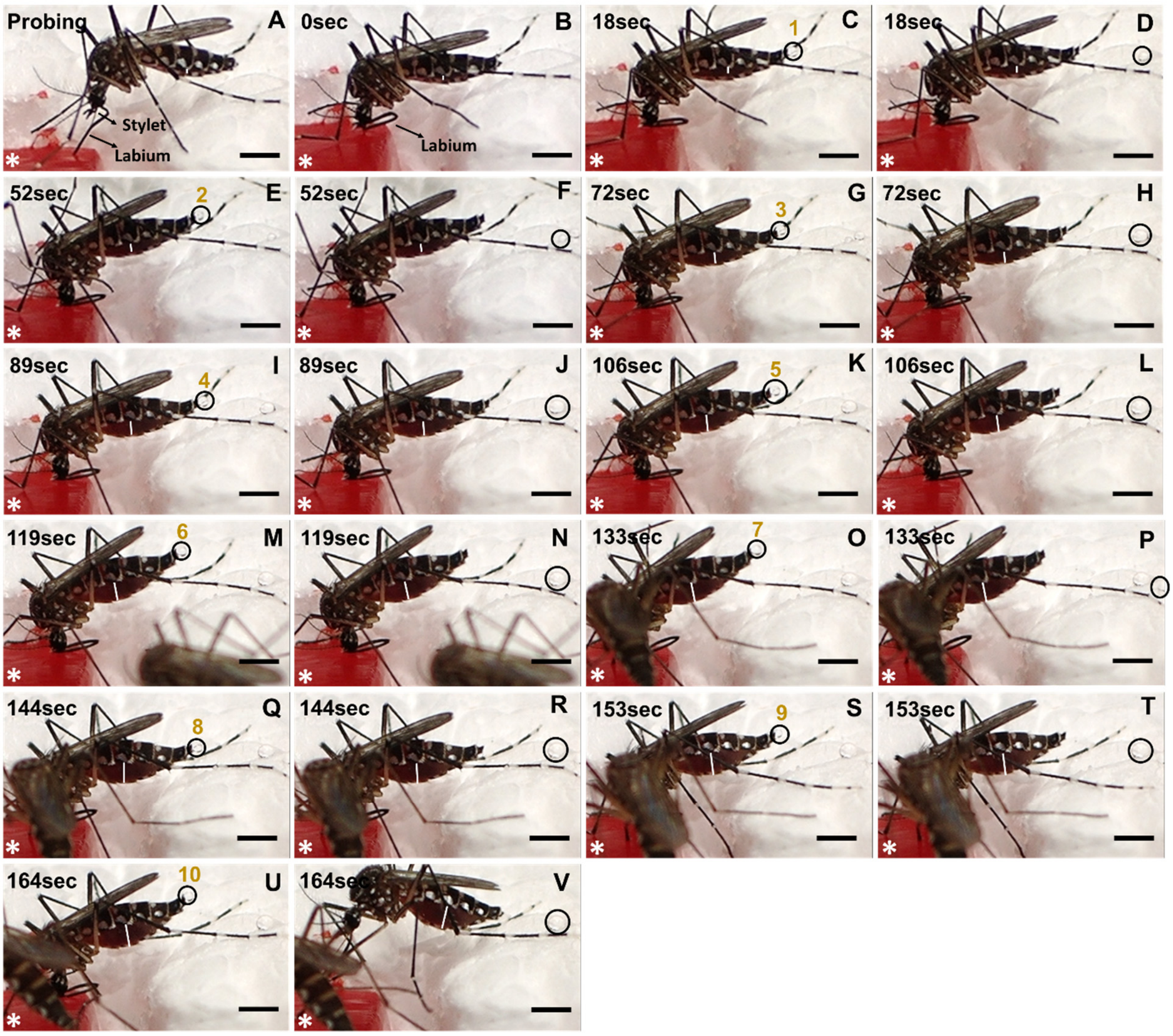

Figure S2: Videography of *Aedes aegypti* female mosquito excreting multiple prediuretic droplets while feeding on blood-loaded Capgel. (**A**) Probing behavior where the mosquito inserts the proboscis into the Capgel (white asterisk) and forages for the desired capillary with stylet mouthparts leaving labium out and bent prior to (**B**) the onset of blood meal (0s). **A–V**) Nonconsecutive, sequential set of images taken from video recordings (**Vid. S3 & S4**) capturing the taking of a blood meal event by an *Ae. aegypti* female from a warmed, blood-loaded Capgel block oriented in raftview: Prediuretic droplets are formed (**C, E, G, I, K, M, O, Q, S, U**) and expelled (**D, F, H, J, L, N, P, R, T, V**), respectively, within a fraction of a second over the course of a 164s (2.73 min) long blood meal. Extension of the abdomen is indicated by a white line across the pleural membrane in each panel. Prediuretic droplets are enclosed by a black circle. Scale bar – 1mm

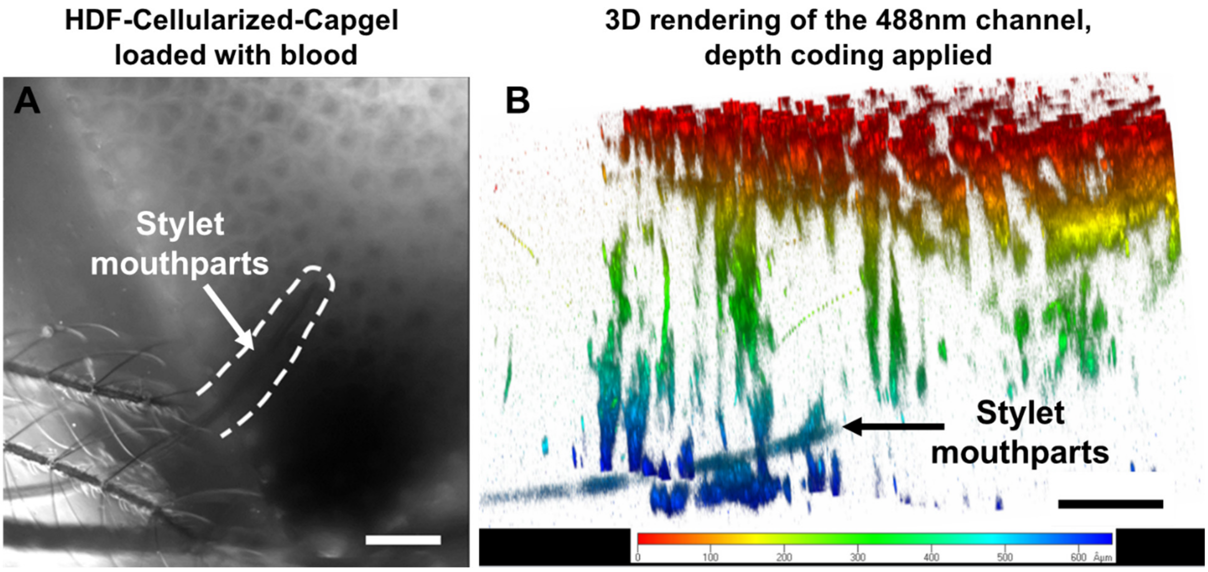

Figure S3: Female *Ae. aegypti* stylet mouthparts penetrate through multiple layers of warmed, blood-loaded HDF BITES microvessel tissue structures. (**A**) Representative DIC micrograph of HDF BITES in capview perspective with stylet mouthparts entering from the raftview side. (**B**) A 3D-rendered image depth coded by 488nm fluorescence of **A,** showing autofluorescence of stylet mouthparts inside BITES. White dashed line outlines the stylet mouthparts within HDF BITES tissue. Scale bar – 200µm

Videos Captions:

Video S1: Red blood cells (RBCs) freely moving/flowing through Capgel capillaries from raftview perspective, mimicking a blood vessel.

Video S2: RBCs freely flowing out of the Capgel capillaries from capview perspective.

Video S3: An *Aedes aegypti* female mosquito feeding on a blood-loaded Capgel. First the mosquito initiates blood feeding by probing, then penetrating the surface of the blood loaded Capgel.

Video S4: Continuation of mosquito feeding on blood-loaded Capgel (video S3). Abdomen size expands due to midgut distention and prediuretic droplet formation is observed.

Video S5: Three-dimensional reconstruction of z-stacks (rendered through the Y-axis), depicting tubular HDF-lined capillaries from the capview perspective. Green: actingreen488, blue: Nucblue.

Video S6: Three-dimensional reconstruction of z-stacks (rendered through the X-axis), depicting tubular HUVEC-lined capillaries from the raftview perspective. Green: actingreen488, blue: Nucblue.

Video S7: Raftview of HDF-cellularized-Capgel with RBCs freely flowing within the capillaries (BITES).

Video S8: Capview of HDF-cellularized-Capgel with RBCs coming out from the ends of the capillaries (BITES).

Video S9: Videorecording of mosquitoes (n=50) showing interest in, probing on and feeding from BITES. This video is a 2 min excerpt taken from the 15min BITES presentation experiment.

Video S10: Videorecording of mosquitoes (n=50) showing interest in, probing on and feeding from BITES. Liquid nitrogen was poured over mosquitoes to freeze stylet mouthparts inside BITES at the end of the video. This video is a 2 min excerpt taken from the 4min BITES presentation experiment.

Video S11: Videorecording of mosquitoes (n=50) showing interest in, probing on and feeding from BITES. This video is a 2 min excerpt taken from the 20min BITES presentation experiment.

Video S12: DIC image of BITES at different focal planes taken from the capview perspective, reveal penetration of stylet mouthparts through many layers of capillaries.

Video S13: DIC image of BITES, same construct in Video S12, at different focal planes through raftview perspective, reveal penetration of stylet mouthparts through many layers of capillaries.

Video S14: DIC images at different focal planes from the raftview perspective of BITES penetrated with stylet mouthparts revealing RBCs visibly inside the labrum.

Video S15: Merged DIC and fluorescence 3D-projection of z-stacks taken from capview orientation of BITES cellularized with HDF, 3-days post-blood-meal (PBM), showing intact capillaries and uncontaminated cell culture.
